# Interoception as a central mechanism in Whole Person Health

**DOI:** 10.1371/journal.pbio.3003487

**Published:** 2025-11-13

**Authors:** Wen G. Chen, Helene M. Langevin

**Affiliations:** National Center for Complementary and Integrative Health (NCCIH), National Institutes of Health (NIH), Bethesda, Maryland, United States of America

## Abstract

Decoding the body’s internal signals could revolutionize Whole Person Health. This Perspective suggests a research framework linking brain, body and behavior to foster resilience, self-regulation and holistic well-being.

## Teaser

Interoception may be a key mechanism in “Whole Person Health.” More and better technologies, tools, and mechanistic research are needed to fully understand its role, as well as new interventions to promote healthy physiological self-regulation through interoceptive signals.

Interoception, the process by which the brain senses, interprets, integrates, and regulates internal bodily signals [1], is not only an emerging and important topic in neuroscience but also a fundamental biological interface central to the health of the whole person [2]. Here, we aim to provide a framework connecting interoception and “Whole Person Health,” highlighting the critical research, technologies, and tools needed to strengthen our understanding and promotion of human health.

In research, the concept of Whole Person Health emphasizes both integration across multiple body systems and positive health processes such as resilience and health restoration [2]. Research on Whole Person Health ranges from basic and mechanistic work on whole body physiology to clinical studies evaluating therapeutic strategies to promote overall health. To enable an easily implementable method to assess Whole Person Health in clinical research, the United States National Center for Complementary and Integrative Health and Centers for Disease Control and Prevention developed the Whole Person Health Index (WPHI), a simple 9-item self-report tool that can be completed in less than 5 min. Respondents rate their self-assessment from 1 (poor) to 5 (excellent) across 9 key domains: overall health, quality of life, social and family connections, healthy diet, physical activity, stress management, sleep quality, sense of meaning and purpose, and health self-management, with higher scores indicating better overall health. Notably, interoception may have a unifying role across all the WPHI domains.

Interoception involves the body’s ability to sense and regulate internal signals such as hunger, heart rate, breathing, immune response, and gut sensation. These signals travel both ways, from organs to the brain and back again, via nerves, hormones, and immune pathways [1]. For example, disruptions in detecting hunger, satiety, and metabolic energy signals can lead to overeating and obesity [3], highlighting the importance of interoception in eating a healthy diet. Interoception is also important in exercise—children with higher interoceptive sensitivity perform better on a 6-min run and engage in more light physical activity in daily life than those with lower interoceptive sensitivity [4]—and in sleep, with poorer sleep quality consistently associated with increased bodily attention but reduced confidence in interpreting bodily signals [5]. In fact, stronger interoceptive abilities are positively linked to lifestyle factors such as exercise, balanced eating, and relaxation practices like mindfulness and meditation, underscoring interoception’s vital connection to overall health and quality of life [6].

Substantial human and animal model data also support a mechanistic bridge between interoceptive signals and stress regulation, including causal evidence linking negative interoceptive signals to avoidance or stress-like responses [7]. In rats, oxytocin-sensitive circuits within the insular cortex, a key interoceptive hub, integrate bodily and socio-emotional cues to either approach or avoid other distressed animals, revealing a neural link between interoception and social affect and connectedness [8]. The anterior insula is thought to have a key role in integrating interoceptive signals to generate subjective feeling and self-awareness; thus, our embodied sense of meaning and purpose may emerge in part from the brain’s integration of bodily states into conscious experience [9].

Many interventions can enhance interoception. Some of them, including meditation, music, and aromatherapy, may act by regulating brain activity through neuro-psychological input. Nutritional input-based interventions such as probiotics, herbal, and dietary supplements would be expected to influence the gut first. Interventions that are primarily physical input-based, such as manual therapies, acupuncture, and thermal therapy, may modulate the musculoskeletal and other internal organ systems first, whereas therapies like yoga, Tai Chi, and other meditative movements may engage both brain and body, plus the pathways in between. To optimize the therapeutic effects of these low-cost, accessible, and nonpharmacological interventions and maximize their impact on Whole Person Health, we need robust tools, technologies, and frameworks that can help measure, map, monitor, and modulate interoception, which we call the 4M Framework.

In this Framework, interoceptive signals, which come in many forms including chemical, electrical, mechanical, thermal, and possibly magnetic or optical, need to be measured precisely within an organism. In both animal model and human research, we now have a growing toolkit, particularly for chemical, electrical, mechanical, and thermal signals. For example, in human studies and relevant animal model research, we can measure blood biomarkers, electroencephalographic activity, heart rate variability, baroreflex, and gut pressure, map responses using PET or fMRI scans and monitor with wearables, while externally modulating with drugs, nutrients, and nerve stimulation, and self-modulating with mind–body therapies. In conjunction with these physiological measurements, the MAIA Scale can be used to measure multidimensional subjective aspects of human interoception in clinical studies [10].

Despite these advances, key challenges remain in implementing the 4M Framework. Real-time monitoring of chemical signals is still limited, making it difficult to capture dynamic physiological changes. Tools for magnetic and optical signals are largely unavailable in humans and underdeveloped in animal models, yet these modalities may carry critical information about our neural and physiological processes, such as magnetoreception or light-sensitive signaling, which remain poorly understood due to the lack of accessible measurement technologies. While animal research offers more advanced tools for manipulating interoceptive signals and circuits, real-time monitoring of chemical, mechanical, and thermal signals also remains difficult. Optogenetics has opened powerful new doors, but its application is mostly restricted to light-based signals in animal models. Thus, enhancing our abilities to measure, map, monitor, and modulate all types of interoceptive signals, especially magnetic and optical signals, may lead to major scientific breakthroughs for interoception research.

**Fig 1:**
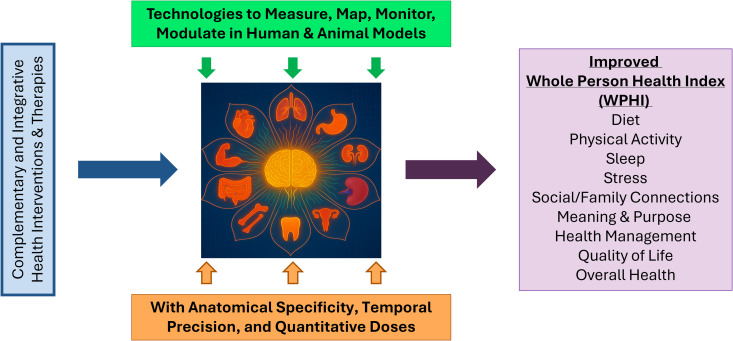
A proposed research model: interoception as the integrative hub for Whole Person Health research. This conceptual model illustrates how interoception functions as a central mechanism linking multisystem interventions to measurable health outcomes. The model positions interoception at the intersection of diverse therapeutic inputs and health domains. Interventions, including psychological, nutritional, and physical, are hypothesized to influence interoceptive signaling either directly or indirectly. These interventions may act on the brain, body, or the bidirectional pathways between them, modulating interoceptive processes in ways that support resilience, stress regulation, and overall well-being. The framework also integrates the 4M Framework (Measure, Map, Monitor, and Modulate) as a scientific strategy to investigate interoception. It includes developing and applying tools to quantify interoceptive signals, map their neural correlates, monitor them in real time, and modulate them through targeted external or internal interventions. By aligning interoceptive mechanisms with the domains of the Whole Person Health Index, the model provides a testable framework for understanding how internal bodily awareness contributes to health outcomes. It also highlights the need for cross-disciplinary research and innovation in measurement technologies, especially for underexplored signal types like magnetic and optical interoception. Ultimately, this model encourages a shift from disease-centered paradigms to a systems-based understanding of health, where listening to the body’s internal signals becomes a foundation for personalized and integrative care. The image was drawn using PowerPoint, and the central flower figure was generated using AI (ChatGPT) with scientific input from Wen G Chen and Helene M Langevin.

To bring it all together, we propose a research model with interoception as a central mechanism in Whole Person Health (Fig 1). By pairing innovative technologies that can measure, map, monitor, or modulate interoceptive signals in humans or animal models with anatomical specificity, temporal precision, and quantitatively optimized dosing, we can begin to study how interventions, especially complementary and integrative health therapies, might enhance humans' ability to self-modulate physiological processes and ultimately improve WPHI outcomes.

If we hope to truly understand and support Whole Person Health, it will start with something fundamentally simple: learning to listen more closely to the body’s own signals. Interoception is a regulatory system, a predictive mechanism, and a therapeutic target. By decoding the body’s internal dialogue, we could unlock a new era of medicine, one that begins not with disease, but with awareness of health.

## Abbreviation

**:**
WPHI: Whole Person Health Index
